# Motor intervention therapy for children with developmental coordination disorder: from behavioral improvement to neuroplasticity mechanisms

**DOI:** 10.3389/fnhum.2026.1757488

**Published:** 2026-05-13

**Authors:** Zhiguang Ji, Liyan Wang, Le Lu, Chenping Zhang, Hongbiao Wang

**Affiliations:** 1 Department of Physical Education, Shanghai University of Medicine and Health Sciences, Shanghai, China; 2 College of Rehabilitation Sciences, Shanghai University of Medicine and Health Sciences, Shanghai, China

**Keywords:** behavioral improvement, brain remodeling, cognitive function, developmental coordination disorder, motor intervention, neuroplasticity

## Abstract

Developmental Coordination Disorder (DCD) is a common neurodevelopmental disorder characterized by significantly delayed motor coordination, often accompanied by cognitive deficits and psychosocial adaptation issues, severely impacting children’s lifelong development. Motor intervention serves as the primary treatment strategy for DCD, encompassing task-oriented, process-oriented, and technology-assisted paradigms. Existing evidence indicates that structured motor interventions effectively enhance motor skills, executive function, and social engagement in children with DCD. The core mechanism lies in inducing neuroplasticity—encompassing both functional reorganization (e.g., normalization of motor network activation, improved inter-regional connectivity) and structural changes (e.g., increased grey matter volume in key brain regions, optimized white matter microstructure). These changes ultimately facilitate behavioral improvement through optimized internal models and enhanced cognitive-motor coupling. This paper constructs an integrated “motor intervention-neuroplasticity-functional improvement” model, reviews intervention efficacy and mechanisms, identifies current research limitations in sample size, causal inference, and long-term follow-up, and outlines future directions such as precision rehabilitation and technology integration. It provides theoretical support for evidence-based interventions in DCD.

## 1 Introduction

Developmental Coordination Disorder (DCD) is a specific neurodevelopmental disorder characterised by impaired motor coordination skills that significantly fall below the expected level for an individual’s age and intellectual ability. These motor difficulties are persistent and severely interfere with academic achievement and activities of daily living (American Psychiatric Association, 2013). According to the criteria outlined in the Diagnostic and Statistical Manual of Mental Disorders, Fifth Edition (DSM-5), the prevalence of DCD among school-aged children is estimated to be approximately 5–6% (Blank et al., 2019). This suggests that almost every classroom may contain one or two children affected by this disorder. To understand how the intervention in movement leads to these changes, the key lies in forming a theoretical framework that links behavior with its neural basis. Among the various cognitive neuroscience models proposed for developmental coordination disorder, the internal model defect hypothesis and the automation defect hypothesis have received substantial empirical support (Wilson et al., 2017a, 2017b; Adams et al., 2014). The internal model defect hypothesis posits that children with developmental coordination disorder have difficulties in forming and using neural representations (internal models) that can predict the sensory outcomes of actions and calculate the necessary motor instructions. This leads them to rely on slow, feedback-based control methods. The automation defect hypothesis is closely related to this, suggesting a core defect in the ability to automatize motor skills through practice, often associated with cerebellar dysfunction. These frameworks are particularly significant for this review because they establish a direct connection between behavioral observations (clumsy, laborious movements) and the potential neural basis (the cerebellar–posterior–anterior circuit that can be detected using modern neuroimaging techniques). Therefore, this review will mainly adopt the concept of internal model optimization and enhanced cognitive-motor coupling (a key element in the automation process) as a perspective for examining and interpreting the evidence of neural plasticity after movement intervention. However, symptoms are often misinterpreted as “clumsiness,” “laziness” or “lack of effort”, resulting in chronic underdiagnosis and delayed intervention in clinical settings (Zwicker et al., 2013; Kumar et al., 2025).

The challenges posed by DCD extend far beyond the difficulties involved in diagnosis; the condition exerts a profoundly negative impact on children’s overall development. In the domain of motor function, affected children exhibit significant challenges in daily activities such as dressing, tying shoelaces, and using utensils. As demonstrated in the research by Barnett et al. (2015), there is a correlation between substandard penmanship and a reluctance to engage in physical activities. This phenomenon serves as a significant barrier to the cultivation of autonomy in daily living skills. From a cognitive perspective, children diagnosed with developmental coordination disorder (DCD) frequently demonstrate deficiencies in executive function, encompassing aspects such as working memory, cognitive flexibility, and inhibitory control (Leonard and Hill, 2014). Additionally, they may encounter challenges in visuospatial processing. These cognitive challenges have been shown to compound academic struggles, particularly in tasks requiring fine motor coordination such as writing and mathematical operations (Cairney, 2015). Within the psychosocial domain, the development of motor skills is of particular significance for peer interaction and participation in play. Consequently, children diagnosed with DCD frequently encounter social isolation and low self-esteem, along with elevated risks of anxiety and depression (Cairney et al., 2013). Longitudinal studies also demonstrate that in the absence of timely intervention, these difficulties persist into adolescence and adulthood, giving rise to a series of “secondary consequences” including low levels of physical activity, increased obesity risk, limited educational achievement, and employment difficulties (Kirby et al., 2014). Consequently, DCD is not merely a motor skill issue, but a significant public health concern impacting children’s lifelong developmental trajectories and quality of life (Blank et al., 2019).

Of particular concern is the high comorbidity rate of DCD with conditions such as Attention-Deficit/Hyperactivity Disorder (ADHD), Autism Spectrum Disorder (ASD), and Specific Learning Disorders (Pranjić et al., 2023; Cacola et al., 2017). This comorbidity has been demonstrated to engender a marked degree of complexity in clinical presentations, thereby significantly increasing the difficulty of intervention. Consequently, elucidating the unadulterated presentation of DCD, in conjunction with its interactions with other disorders, is imperative for the provision of precise assessments and support.

In view of the core deficits and extensive impact of DCD, the early identification and effective intervention of this condition are of particular importance. In the field of intervention approaches, motor intervention is a widely recognised primary strategy and core approach for managing DCD (Blank et al., 2012). The theoretical rationale is clear: systematic, structured motor training directly addresses the weaknesses of children with DCD, promoting motor skill development, improving physical condition, and enhancing their confidence in participating in activities. Current common motor intervention paradigms can be categorised into two main classifications. Firstly, there is the “bottom-up” process-oriented intervention, which focuses on improving foundational abilities (e.g., sensory integration, basic muscle strength, and endurance). Secondly, there is the “top-down” task-oriented intervention, which includes the Cognitive Orientation to daily Occupational Performance (CO-OP) method. This method focuses on teaching children problem-solving strategies within functional tasks (Martini et al., 2014). Moreover, with technological advancements, emerging technologies such as virtual reality (VR) and active video games (AVG) have provided new delivery platforms for motor interventions (Jane et al., 2018).

Evidence from randomized controlled trials and systematic reviews indicates that well-structured exercise interventions can significantly improve motor skills in children with DCD in the short term (e.g., scores on the Movement Assessment Battery for Children, Second Edition [MABC-2]) and enhance their motivation to participate in activities (Wilson et al., 2013). However, a notable phenomenon in clinical practice and research is the heterogeneity of intervention outcomes: responses to the same intervention can vary significantly among children (Sugden, 2007). This heterogeneity may be attributable to some factors, including different subtypes of DCD, varying comorbidities, individual cognitive characteristics, or differences in environmental support.

However, a critical gap persists in current DCD motor intervention research: the majority of studies remain confined to a “black box” input–output model. The primary focus of these studies is on identifying which interventions are effective for which children, while the underlying biological mechanisms driving intervention efficacy are not sufficiently explored. This delay in mechanistic research imposes significant constraints on the optimization of interventions and hinders progress toward personalized, precise rehabilitation. In a manner analogous to the progression of medicine from a “symptom-based treatment” model to a “cause-based treatment” paradigm, it is imperative to investigate the mechanisms underlying DCD motor interventions to transition from an “empirical” to a “mechanism-driven” approach (Zwicker et al., 2009). There is an urgent necessity to address a series of critical scientific questions: The present study aims to explore the impact of motor training on the brain structure and function of children diagnosed with DCD. The question, therefore, arises as to how these neuroplastic changes correlate with Behavioral improvements. The central question guiding this study is whether different intervention methods, such as CO-OP and sensory integration training, operate through distinct neural pathways. The provision of answers to these questions is of significant theoretical value, insofar as it offers unique insights into the interaction between motor skill learning and brain development. Furthermore, the questions hold urgent clinical relevance, insofar as they provide scientific foundations for the development of more efficient and targeted rehabilitation programmes.

In consideration of the aforementioned, the objective of this review is twofold: firstly, to methodically organise and synthesise extant research evidence on the efficacy of motor interventions for DCD; and secondly, to undertake an in-depth exploration of the underlying Behavioral and neuroplasticity mechanisms. The paper sets out three specific objectives. Firstly, the Behavioral efficacy must be systematically evaluated by means of a comprehensive review and synthesis of evidence from a range of movement interventions (including task-oriented, process-oriented, and technology-assisted approaches) about improvements in core motor skills, executive function, engagement, and quality of life among children diagnosed with DCD. This encompasses the analysis of critical factors that influence outcomes, such as intervention dosage, intensity, and individual variability. Secondly, the aim is to elucidate underlying neural mechanisms, with a focus on integrating recent findings from neuroimaging techniques (e.g., functional MRI [fMRI], structural MRI [sMRI], diffusion tensor imaging [DTI]) and neurophysiological methods. This will systematically elucidate how motor interventions induce plasticity changes in the brains of children with DCD, including functional reorganization in key brain regions (e.g., cerebellum, basal ganglia, prefrontal cortex), enhanced neural network connectivity efficiency, and improved white matter microstructure. Thirdly, the establishment of connections and the delineation of future directions is facilitated by the construction of a relational model that links “motor intervention-neuroplasticity-behavioral improvement.” In light of the prevailing research gaps and limitations, we put forward some promising avenues for future exploration with a view to advancing DCD rehabilitation practices toward a new paradigm grounded in evidence and mechanisms.

This review covers a wide range of topics (aiming to integrate evidence regarding behavioral outcomes and neural plasticity mechanisms), and we have adopted a narrative review approach. This method is suitable for integrating results from various research designs (such as randomized controlled trials, neuroimaging studies, and systematic reviews) to construct a comprehensive theoretical model. To ensure the comprehensiveness and systematic nature of the literature base, we conducted a structured search of electronic databases, including PubMed, PsycINFO, and Web of Science, up to articles published in December 2025. The search strategy combined terms related to the research subjects (“developmental coordination disorder” or “DCD”), intervention measures (“motor intervention” or “task-oriented training” or “CO-OP” or “virtual reality”), and outcomes (“neural plasticity” or “brain imaging” or “functional magnetic resonance imaging” or “diffusion tensor imaging” or “motor skills”). We prioritized the inclusion of high-quality evidence, such as systematic reviews, meta-analyses, and randomized controlled trials, while also considering relevant longitudinal neuroimaging studies and theoretical papers to supplement the discussion of mechanisms. In the following sections, we will present these findings in a narrative manner, focusing on key themes and integrating various findings to construct the model we have proposed.

## 2 Clinical heterogeneity of DCD and its neurological basis

In order to gain a deeper understanding of the impact of exercise intervention on Developmental Coordination Disorder (DCD), it is essential to comprehensively grasp the clinical characteristics of DCD itself and its underlying neurobiological basis. DCD is not a single disease but a complex spectrum, showing significant heterogeneity in symptom manifestations, comorbidity patterns, and potential neural mechanisms. This review will systematically outline the core deficits of DCD, as well as the intrinsic heterogeneity of the disorder and the neural associations revealed by current neuroimaging studies. This will establish a solid foundation framework for subsequent discussions on the behavioral and neural changes triggered by exercise intervention.

### 2.1 Core movements and cognitive deficits

The most prominent feature of Developmental Coordination Disorder (DCD) is the presence of widespread motor coordination impairments. These impairments are not directly caused by intellectual disabilities, identifiable neurological diseases, or other physical disabilities, but stem from the dysregulation of neural functions during motor control and learning processes (American Psychiatric Association, 2013).

Children with DCD face difficulties in many areas. For fine motor skills, they struggle with tasks that need good hand-eye coordination and dexterity. Their handwriting is often slow and hard to read. Simple daily tasks like using utensils, buttoning shirts, or tying shoelaces can also be challenging (Chang and Yu, 2010; Barnett et al., 2015). Gross motor skills are also affected. Children with DCD have trouble with running, jumping, and balancing during movement. Research suggests this happens because they rely too much on vision to control their posture. They also have trouble integrating information from their body’s senses (Deconinck et al., 2006; Ferguson et al., 2014). Learning new movements is another area of difficulty. Children with DCD struggle to learn and execute new movement sequences. This reflects problems with procedural learning, the kind of learning that helps skills become automatic (Wilson et al., 2013). They also perform poorly on motor imagery tasks, such as mentally rotating objects, suggesting they have trouble creating and using internal models of movement (Adams et al., 2014). Coordination between limbs is also problematic. When catching a ball or doing rhythmic movements, children with DCD show more variability in timing and space. Their movements are less stable, making it hard to establish consistent coordination patterns (Volman et al., 2006). Beyond movement itself, executive function is often impaired. Children with DCD commonly have trouble with working memory, especially for visual–spatial information. They also struggle with cognitive flexibility and inhibitory control (Leonard et al., 2015; Bernardi et al., 2018). These challenges may explain why they find it hard to handle complex motor situations that require processing multiple things at once or quickly changing strategies (Lachambre et al., 2021). Visual–spatial processing is another area of weakness. Some children with DCD have trouble judging spatial relationships between objects. They may find it hard to pick out information from a busy background or remember what they have seen (Wilson and McKenzie, 1998). These difficulties affect real-time movement adjustments, such as reaching for an object or catching something moving toward them (Coetzee and Pienaar, 2010). Finally, many children with DCD have problems with sensory processing. They may overreact or underreact to touch, movement, or body position cues. They also have trouble combining information from different senses (Zwicker et al., 2009). These issues can interfere with developing an accurate sense of where their body is in space, which then affects how they plan and carry out movements.

### 2.2 Subtypes and comorbidity

DCD exhibits significant clinical heterogeneity. Cluster analysis studies have classified patients into different subtypes based on patterns of visual-motor integration, proprioception, or motor control impairments (Dewey and Kaplan, 1994). Clinical observations also indicate that subgroups with additional conditions (such as joint hypermobility syndrome) may exhibit more extensive neurodevelopmental features, including attention disorders and language problems, suggesting that lax connective tissue may have a broader impact on neurodevelopment (Adib et al., 2005; Kirby and Davies, 2007).

In addition to this inherent heterogeneity, DCD often co-occurs with other neurodevelopmental disorders, which can be classified into three major categories. Firstly, attention deficit/hyperactivity disorder (ADHD) is the most common comorbidity; children with both of these disorders face a higher risk of psychological distress than those with only one disorder (Piek et al., 2007). Secondly, a significant number of individuals with autism spectrum disorder (ASD) also exhibit motor coordination difficulties that meet the diagnostic criteria for DCD, with a significant overlap in the domains of motor planning and executive function (Bhat, 2020). Thirdly, specific learning disorders such as dyslexia often co-occurs with DCD, which may reflect a common neural basis, including cerebellar function (Kaplan et al., 1998). This complex comorbidity situation suggests the existence of common neural pathways, such as dysfunction of the cerebellum-frontal circuit, and emphasizes the importance of developing personalized assessment and intervention strategies (Nicolson and Fawcett, 2011).

### 2.3 Preliminary evidence for neurobiological foundations

The advancements in neuroimaging research mainly provide empirical evidence for the systematic structural and functional differences between the brains of children with developmental coordination disorder (DCD) and their normally developing peers.

Structural abnormalities: The cerebellum is traditionally associated with motor coordination and balance functions. Children with DCD have a reduced volume or structural abnormalities in specific areas of the cerebellum, such as the vermis (Brown-Lum and Zwicker, 2015). Diffusion tensor imaging (DTI) studies have shown a decrease in fractional anisotropy (FA) values of the corpus callosum, indicating possible myelin formation defects or poor white matter fiber organisation, which may be the cause of difficulties in limb coordination (Brown-Lum et al., 2020). Additionally, a reduction in grey matter volume or changes in cortical thickness are observed in the parietal cortex (involved in sensory integration and motor planning) and the prefrontal cortex (responsible for advanced motor planning and cognitive control) (Langevin et al., 2015).

Functional abnormalities: Functional magnetic resonance imaging (fMRI) studies show abnormal brain activation patterns during the execution of motor tasks. Children with DCD tend to rely excessively on frontal lobe regions related to conscious control, such as the dorsolateral prefrontal cortex, while there is insufficient activity in the circuits responsible for automatic movements (including the cerebellum and basal ganglia) (Kashuk et al., 2017). This pattern indicates lower efficiency in processing motor information and the need for greater cognitive effort to complete motor tasks. Resting-state fMRI studies further reveal abnormal functional connectivity between the default mode network (DMN) and task-positive networks (such as the sensory-motor network), which may hinder the ability to rapidly transition from a resting state to task engagement (McLeod et al., 2014). Supplementary electroencephalogram (EEG) studies have revealed abnormal neural oscillation activities. Children with DCD exhibit weaker middle frontal theta oscillations in tasks requiring concentration, which is associated with poorer performance (Wang et al., 2015).

Overall, the neurobiology of developmental coordination disorder involves a wide network, including the cerebellum, basal ganglia, sensory-motor cortex, parietal lobe, and prefrontal lobe, with subtle structural abnormalities and functional coordination failures, leading to core deficits in motor planning, execution, learning, and coordination. Current theoretical models, including the internal modeling deficit hypothesis (which suggests difficulty in forming neural representations that predict the impact of movements on sensory inputs) and the automation deficit hypothesis (which suggests that motor skills cannot be automated through practice), aim to integrate these neuroand behavioral findings (Wilson et al., 2017a, 2017b). Understanding this neural basis is crucial for researching how motor interventions can induce neural plasticity and promote behavioral improvement.

## 3 Behavioral efficacy of exercise interventions: a systematic review of evidence

In the preceding two decades, research on exercise interventions for developmental coordination disorder (DCD) has advanced considerably, giving rise to a variety of theoretical orientations and practical paradigms. It is imperative to methodically categorise these intervention paradigms and to undertake a rigorous examination of their theoretical underpinnings. Furthermore, a comprehensive evaluation of their efficacy in enhancing motor skills, cognitive function, and psychosocial adaptation—through a critical review of extant literature (particularly systematic reviews and meta-analyses)—alongside an in-depth exploration of key factors influencing intervention outcomes, holds substantial significance.

### 3.1 Classification of intervention paradigms and theoretical orientations

Interventions for DCD can be categorised into three main types based on their theoretical orientation and focus: task-oriented interventions, process-oriented interventions, and integrated and emerging interventions. Each of these orientations is predicated on a distinct conception of the etiology of DCD, giving rise to a range of intervention strategies.

#### 3.1.1 Task-oriented interventions

The fundamental principle underpinning task-oriented interventions is predicated on the notion of prioritising functionality. Rather than focusing on the remediation of “potential” underlying sensorimotor deficits, the emphasis is placed on facilitating the performance of functional tasks and daily activities that are desired or required by the child (Polatajko and Cantin, 2005). The theoretical foundation of this approach is primarily rooted in learning theory and cognitive psychology.

Among these, the Cognitive Orientation to Daily Occupational Performance (CO-OP) method is considered to be one of the most representative and evidence-supported approaches within task-oriented intervention. This therapist-guided approach is centred on the child’s needs, with the explicit teaching of the “Global Strategy”—specifically the “Goal-Plan-Do-Check” cycle—to address motor challenges encountered in daily life (Anderson et al., 2017). During intervention, therapists assist children in establishing specific and meaningful objectives (e.g., “learn to ride a bike” or “write more legibly”). They then guide children to devise their own plans (e.g., “I’ll sit steady, pedal with my feet, and look ahead”), execute the plan, review outcomes, and make adjustments for improvement. CO-OP places particular emphasis on children using cognitive strategies and self-talk guidance to enhance their metacognitive abilities and self-regulation, empowering them to actively master skill learning. The extant research indicates that CO-OP is efficacious not only in enhancing performance on target tasks but also in extending its effects to a certain degree to analogous untrained tasks (Cantin et al., 2024).

Another approach is Neuromotor Task Training (NTT), which also falls under the task-oriented paradigm but focuses more on enhancing motor learning by adjusting task demands and environmental conditions. The theoretical foundation of this approach is dynamic systems theory, which posits that movement generation is the result of the interaction between the child, task requirements, and the environment (Ferguson et al., 2013). In the field of sensory rehabilitation, therapists tend to eschew overly segmented sensory training methods, which are often characterised by tedium. Instead, they design meaningful, real-life activities, such as walking on surfaces of varying textures, catching balls of varying sizes and speeds. By adjusting task difficulty (e.g., shortening distances, slowing speeds) and environmental settings (e.g., adding handrails, enlarging target zones), therapists assist children in completing tasks. This enables the subjects to discover and refine movement patterns through interaction with their environment.

The merits of task-oriented intervention are evident: it directly addresses the functional challenges that are of utmost importance to children and parents, motivates strong participation, and yields easily observable and measurable outcomes. Consequently, numerous international clinical guidelines advocate its utilisation as the primary intervention strategy for DCD (Blank et al., 2019).

#### 3.1.2 Process-oriented interventions

The fundamental premise of process-oriented interventions is that the motor difficulties experienced by children with DCD stem from deficits in one or more underlying sensorimotor processes (e.g., sensory integration, visuomotor integration, or proprioception). The intervention’s objective is thus evident: to “repair” or enhance these underlying processes through repetitive, intensive practice, thereby aiming for a “bottom-up” improvement in overall motor skills (Schoemaker and Smits-Engelsman, 2015). Within this category, the two most common approaches are Sensory Integration Therapy and Perceptual-Motor Training.

##### 3.1.2.1 About sensory integration therapy

Theory postulating the existence of a Specific Intellect (SI) suggests that a multitude of learning and Behavioral challenges emanate from the brain’s inefficiency in adequately integrating and processing sensory information from the body and environment (American Academy of Pediatrics, 2012). For DCD, SI therapy provides rich, controlled sensory stimulation (e.g., vestibular, proprioceptive, tactile) through challenging play activities. This facilitates the child’s nervous system in the more effective organisation and integration of sensory information, which in turn improves postural control, motor planning, and Behavioral organisation. Examples of such activities include the utilisation of suspension equipment and the exploration and engagement in play activities within designated sand pits.

##### 3.1.2.2 Perceptual-motor training

This approach generally comprises a series of structured exercises meticulously designed to enhance fundamental abilities such as visual perception, proprioception, eye-hand coordination, balance, and rhythm, either as standalone exercises or in combination. Specifically, the programme incorporates activities such as walking on balance beams, tracing patterns, catching and throwing balls, and moving to rhythmic cues (Homanian and Ebrahimi, 2022).

Nevertheless, the effectiveness of process-oriented interventions remains a subject of debate. A plethora of systematic reviews and meta-analyses have been conducted, and while some studies have demonstrated positive effects on specific motor skills (e.g., balance), there is a paucity of robust evidence to demonstrate that training underlying processes effectively and broadly translates to functional activities and daily participation (Ferguson et al., 2013; Smits-Engelsman et al., 2013). For instance, a study examining perceptual-motor interventions for first-grade children with DCD found that while balance abilities significantly improved, overall motor skills and hand dexterity did not show consistent gains (De Milander et al., 2015). Consequently, contemporary evidence-based practice increasingly recommends task-oriented interventions.

#### 3.1.3 Comprehensive and emerging interventions

Recent technological advancements, coupled with a growing comprehension of the intricacies involved in DCD, have collectively led to the progressive development of comprehensive and emerging intervention models. The majority of these models seek to integrate the strengths of different intervention paradigms or leverage new technologies to enhance engagement and effectiveness.

With regard to integrated interventions, a significant proportion of contemporary programmes no longer adhere to a singular, task-oriented, or process-oriented approach. Instead, there has been an adoption of a combined (or hybrid) model. For instance, an intervention plan might be developed around the CO-OP framework, while incorporating specific balance exercises or sensory integration activities that have been tailored to address the child’s individual challenges. This model emphasizes the individualisation and flexibility of intervention, eschewing a one-size-fits-all approach.

Beyond integrated approaches, technology-based interventions such as virtual reality (VR) and active video games (AVG) are gaining prominence. Systems such as the Nintendo Wii Fit and the Xbox Kinect provide highly engaging platforms for motor interventions. These technologies offer a combination of immediate visual and auditory feedback, along with the creation of safe, controllable, and challenging virtual environments. This, in turn, has been shown to significantly boost children’s engagement (Bonnechère et al., 2016). Research also indicates that AVGs, particularly motion-sensing systems, show promising and significant effects in improving balance abilities in children with DCD (Piñar-Lara et al., 2025). Nevertheless, extant evidence pertaining to the efficacy of AVG in relation to other motor skills, including ball skills and hand dexterity, remains inadequate. Furthermore, the extent to which AVG surpasses traditional interventions requires confirmation through additional high-quality studies (Smits-Engelsman et al., 2018). Furthermore, the findings suggest that the movement patterns exhibited by children diagnosed with Developmental Coordination Disorder (DCD) during Active Video Game Play (AVG) differ from those of typically developing children. This suggests that when selecting AVG as an intervention tool, careful consideration must be given to whether the quality of movement aligns with therapeutic goals (Gonsalves et al., 2015).

Furthermore, interventions targeting physical fitness and fundamental motor skills have become a key focus for children diagnosed with DCD. The majority of children diagnosed with DCD demonstrate deficiencies in key physical fitness domains, including cardiorespiratory endurance and muscular strength, in addition to evident deficits in fundamental motor skills (FMS). Consequently, the implementation of specialised training programmes focusing on enhancing physical fitness and fundamental motor skills is imperative. For instance, research demonstrates that fundamental motor skills training effectively enhances both FMS proficiency and self-perception of physical abilities in children with DCD (Farhat et al., 2015). In a similar vein, while core stability training and task-oriented training have been shown to have comparable effects on improving gross motor skills, the latter may offer a more pronounced advantage in enhancing balance control (Au et al., 2014).

### 3.2 Systematic evaluation of efficacy

By referring to the research findings of systematic reviews and meta-analyses published over the past two decades, we can form a comprehensive and quantifiable understanding of the behavioral effects of exercise intervention measures for DCD patients. It is important to note that this review does not conduct a new meta-analysis. Instead, it integrates and summarizes the quantitative results (such as effect sizes) reported in previous high-quality systematic reviews and meta-analyses in a narrative manner, serving as evidence for the intervention effects.

#### 3.2.1 Improvement in motor skills

In general, exercise interventions have been shown to have moderate to large effect sizes in enhancing motor skills in children diagnosed with Developmental Coordination Disorder (DCD). An early landmark systematic review and meta-analysis covering studies from 1995 to 2011 found that task-oriented interventions and traditional physical or occupational therapy significantly improved motor performance, whereas process-oriented interventions yielded weaker effects (Smits-Engelsman et al., 2013). This conclusion was further corroborated by a more recent meta-analysis (Gao et al., 2025).

About the broader concept of motor competence, a meta-analysis of studies conducted between 2012 and 2017 revealed that activity-oriented interventions—which bear a notable similarity to task-oriented approaches—in conjunction with physical function-focused interventions, exhibited a significant positive impact on the motor function and skills of children diagnosed with DCD (Jane et al., 2018). A more recent meta-analysis also provided specific data: motor skill interventions significantly improved overall motor skills in children with DCD (*g* = 1.00, 95% CI [0.48, 1.52], *p* < 0.001) and markedly enhanced balance function (*g* = 0.57, 95% CI [0.17, 0.97], *p* = 0.005; Gao et al., 2025).

However, it is important to note that intervention effects may vary across different skill domains. In the context of balance abilities, a range of intervention strategies, including AVG and physical training, have been shown to yield positive outcomes. Conversely, enhancements in hand-eye coordination and writing skills are more likely to be achieved through highly targeted cognitive-task interventions such as CO-OP (Cantin et al., 2024). In the context of more complex coordination tasks, such as those involving ball skills, it is often necessary to engage in more extensive and targeted training to achieve substantial improvements.

#### 3.2.2 Cognitive and psychosocial benefits

The benefits of exercise interventions extend far beyond the scope of physical activity. A mounting body of evidence suggests that effective exercise programmes can yield a wide range of spillover effects, exerting a favourable influence on both cognitive and psychosocial domains.

With regard to executive function, as previously mentioned, DCD frequently occurs in conjunction with deficits in executive function. Research indicates that children participating in CO-OP interventions demonstrate significant improvements in self-regulation (a core component of executive function), and this improvement serves as a key mediating factor for their enhanced motor performance (Jokić et al., 2013). Furthermore, interventions targeting fundamental motor skill development have been found to enhance inhibitory control in preschool children (Bai et al., 2022), thus supporting the notion of “co-development of motor and cognitive skills.”

Beyond the realm of cognitive enhancements, physical interventions have been demonstrated to exert a favourable influence on the psychological wellbeing and self-perception of children, with a particular emphasis on self-efficacy and body self-concept. The successful mastery of motor skills has been demonstrated to significantly boost children’s self-efficacy, defined as their belief in their ability to accomplish a task. Research indicates that the low levels of physical activity observed in children diagnosed with DCD are primarily attributable to diminished generalised self-efficacy toward physical activities (Cairney et al., 2010). This construct encompasses confidence, preference for movement, and enjoyment. Consequently, it is imperative to enhance their motor abilities through intervention to increase successful experiences, thereby disrupting the detrimental cycle of “failure-avoidance-diminished ability.”

This enhancement in self-perception has been demonstrated to extend to mental health and social participation. Children diagnosed with DCD are already at elevated risk of developing internalising symptoms, such as anxiety and depression. The implementation of effective interventions has been demonstrated to result in a substantial reduction in psychological distress. For instance, group gaze training has been demonstrated to enhance children’s throwing and catching skills, as well as to engender significant improvements in confidence, social skills, and interest in physical activities, as reported by parents (Wood et al., 2017). Moreover, enhanced motor skills have been demonstrated to directly enhance social engagement. Specifically, increased participation in recess games, physical activities, and peer interactions has been shown to result in a reduction in feelings of isolation, thereby leading to a comprehensive improvement in quality of life (Piek et al., 2006).

#### 3.2.3 Influencing factors: moderators of treatment effectiveness

The outcomes of motor intervention are not predetermined; rather, they are influenced by a multitude of factors, including the characteristics of the intervention itself, individual differences among children, and environmental factors. Dosage is a critical factor in determining effectiveness. Dosage is chiefly concerned with the frequency of intervention (e.g., number of sessions per week), the intensity (duration and difficulty of each training session), and the total duration. Specifically, it has been demonstrated that a higher frequency and greater intensity of training generally yield better outcomes. For instance, one study found that a 12-week group motor skills intervention conducted twice weekly significantly improved motor abilities in children with DCD; however, interventions conducted only once weekly with fewer total sessions showed less pronounced effects (Caçola et al., 2016). Furthermore, short-term interventions (for example, 8–12 weeks) have been shown to yield significant outcomes; however, the maintenance of these effects over an extended period may necessitate ongoing support or subsequent “booster” interventions.

It is evident that, in addition to the intervention dosage, the outcomes of the intervention are influenced by the characteristics of the children themselves. With regard to age, early intervention is widely regarded as being more efficacious. The initiation of intervention during the preschool or early elementary school years can facilitate the compensation of deficits experienced during critical periods of motor skill development. This, in turn, can prevent subsequent psychological and social issues. However, intervention remains effective even for older children or adolescents, though greater attention to their motivation for participation and the setting of individualized goals tailored to their needs may be required. Concerning the severity of symptoms and their subtypes, it is acknowledged that children with different subtypes of DCD may respond differently to various interventions. For instance, children with pronounced visuospatial processing difficulties may benefit more from interventions incorporating visual support strategies. This underscores the necessity for flexible intervention plans to accommodate such individual variations. Furthermore, in practical operations, children with other comorbidities (such as attention deficit disorder or autism spectrum disorder) often make the intervention process much more complicated. Therefore, a comprehensive multidisciplinary approach is necessary. At the same time, the existing research evidence is still not deep enough and has not fully explored how comorbidities regulate the intervention effect. The attention deficit of children with attention deficit disorder may hinder their participation in highly cognitive intervention activities, such as the CO-OP intervention process, thereby affecting the participation level and ultimately leading to an increase in the intervention dosage or the need for additional attention-specific training. Similarly, the social communication difficulties exhibited by children with autism spectrum disorder also affect their participation in group exercise interventions. More importantly, whether the neural plasticity changes in children with pure developmental coordination disorder are different from those in children with comorbid attention deficit disorder or autism spectrum disorder remains an unsolved mystery. To truly explain these aspects, future research efforts should design controls to compare the responses of different child subgroups to intervention measures, and no longer consider developmental coordination disorder as a homogeneous and undifferentiated whole. Thus, research can explore neural pathway differences and formulate targeted intervention strategies.

It is imperative to consider environmental factors in addition to the intervention itself and the child’s individuality. The implementation and structuring of interventions across multiple settings – clinical (e.g., one-on-one or group), school, and home–is of crucial importance. Collaborative home-schooling has been demonstrated to be a particularly efficacious strategy, enabling children to apply acquired skills in a variety of contexts and maintain intervention outcomes. For instance, in CO-OP or home-based AVG interventions, parental involvement has been shown to significantly enhance outcomes (Reynolds et al., 2024). The provision of social support from parents, teachers, and peers has been demonstrated to be a crucial motivator, encouraging children to demonstrate persistence in interventions and in the practice of the skills they are attempting to acquire. Furthermore, the cultivation of a favourable, inclusive environment—as opposed to one characterised by criticism or exclusion—is imperative for the realisation of the psychosocial benefits of intervention.

To summarise, the extant literature indicates that systematic evidence demonstrates the efficacy of structured motor interventions, particularly task-oriented and cognitive strategy-based approaches such as CO-OP, in enhancing core motor skills in children diagnosed with DCD. These interventions have also been shown to engender broad cognitive and psychosocial benefits. The efficacy of these interventions is not a matter of chance; it is predicated on the provision of adequate dosage, the creation of a bespoke programme design, and the comprehensive utilisation of environmental support. However, a crucial question arises: The purpose of this study is to ascertain the underlying mechanisms behind the substantial Behavioral changes that have been observed. The central question guiding this study is whether these alterations involve plastic changes in brain function and structure. This will be the core topic explored in depth in the next chapter—the neuroplasticity mechanisms of motor interventions.

## 4 From behavioral improvement to brain remodeling: elucidating mechanisms of neuroplasticity

Significant Behavioral improvements are invariably accompanied by dynamic changes in brain function and structure. The objective of this chapter is twofold: firstly, to transcend Behavioral observations and, secondly, to delve into the neuroscientific level to systematically elucidate how motor interventions induce positive plasticity changes in the brains of children with DCD. In accordance with a “function–structure-mechanism” logical sequence, the present study will firstly review evidence of plasticity at the functional level (including task-state and resting-state fMRI), then explore alterations at the structural level (grey matter and white matter), and finally integrate these findings to propose a cognitive-neural mechanism framework explaining how motor intervention optimises the “internal model” and enhances “cognitive-motor coupling” in children with DCD.

### 4.1 Plasticity at the functional level

The utilisation of functional neuroimaging techniques, with a particular emphasis on functional magnetic resonance imaging (fMRI), offers a unique opportunity to observe the cognitive processes of children diagnosed with developmental coordination disorder (DCD). This approach provides a comprehensive perspective, capturing changes in brain function both before and after intervention. The results of these studies demonstrate that intervention-induced alterations in brain function result in reorganization and optimization.

#### 4.1.1 Task-state fMRI evidence: normalization and automation of motor control networks

Resting-state fMRI studies are a method of directly observing brain activation patterns by scanning children during specific motor tasks (e.g., finger tapping sequences, imagined movements, balance control). In contrast to typically developing (TD) children, DCD children demonstrate significantly abnormal brain activation patterns during motor tasks, primarily characterised by inefficient neural network recruitment (Bonthrone et al., 2024). The primary function of motor intervention is to facilitate the transformation of these abnormal activation patterns toward a state of normality and enhanced efficiency.

The most critical manifestation of this transformation is the normalization of activation within the prefrontal-cerebellar-parietal network. Children diagnosed with DCD frequently demonstrate an excessive reliance on activation in the prefrontal cortex (PFC), particularly the dorsolateral prefrontal cortex (DLPFC), during the execution of motor tasks (Zwicker et al., 2011). The DLPFC is a key brain region for higher-order cognitive control (e.g., working memory, cognitive flexibility). This overactivation is interpreted as a compensatory mechanism: due to impaired function in the cerebellum-basal ganglia-motor cortex circuit responsible for automated movement execution, children with DCD must rely more heavily on conscious, effortful cognitive control to “think through” how to perform actions (Kashuk et al., 2017). This “top-down” control pattern is inefficient, resulting in clumsy, slow, and fatigued movements.

Following the implementation of effective motor interventions, there was a marked improvement in the abnormal activation pattern, with three specific changes being observed. Firstly, it has been demonstrated that excessive prefrontal activation diminishes. As motor skills become proficient, reliance on conscious control decreases, significantly reducing overactivation in regions such as the DLPFC (Irie et al., 2021). This finding suggests a transition from effortful cognitive control to more automated movement execution. Secondly, an increase in cerebellar and basal ganglia activation has been observed. Concurrently, activation levels in the cerebellar and basal ganglia regions (e.g., the putamen)—which are closely linked to motor automation, temporal control, and error detection—normalize or enhance (Zwicker et al., 2011). This finding is indicative of enhanced function in these core motor brain areas, leading to improved movement fluency and precision. Thirdly, the parietal cortex is observed to function at an optimised level. The parietal cortex, and more specifically the inferior parietal lobule, has been shown to govern sensorimotor integration and spatial representation. Activation patterns in this region are frequently abnormal in children diagnosed with DCD. Subsequent to intervention, these patterns become more analogous to those exhibited by typically developing (TD) children, thereby signifying enhanced integration efficiency between sensory information and motor commands (McLeod et al., 2014).

Collectively, these changes point to a core conclusion: motor intervention promotes the automation of motor control in children diagnosed with DCD. At the neural level, this manifests as reduced engagement of the Frontoparietal Control Network (FPCN) during simple or learned motor tasks. As a fundamental element of the FPCN, the prefrontal cortex plays a pivotal role in regulating brain activity during cognitively demanding tasks (Cairney, 2015). Research findings indicate that adolescents diagnosed with DCD demonstrate excessive prefrontal activation during motor tasks, indicative of diminished motor automation and augmented reliance on cognitive control (Al-Yahya et al., 2023). Consequently, the diminished prefrontal activation detected subsequent to motor intervention can be construed as a neural indicator of progress toward motor skill automation, denoting reduced reliance on deliberate cognitive control (Al-Yahya et al., 2023; Wilson et al., 2017a, 2017b). This automation has been demonstrated to enhance motor efficiency, while concomitantly freeing up cognitive resources. This has been shown to result in an improvement in executive function following intervention.

#### 4.1.2 Resting-state fMRI evidence: enhanced brain network connectivity efficiency

Resting-state fMRI (rs-fMRI) is a neuroimaging technique that quantifies spontaneous neural activity in the brain during a task-free, resting state. The analysis of spontaneous activity across different brain regions (i.e., functional connectivity) provides insights into the organisational efficiency of the brain’s intrinsic functional networks. Children diagnosed with developmental coordination disorder (DCD) also exhibit abnormalities in functional connectivity during rest. Intervention has been shown to promote optimization of network efficiency in this group.

This optimization is first manifested in enhanced connectivity within the sensorimotor network (SMN). The SMN, comprising the primary motor cortex, primary somatosensory cortex, and supplementary motor area, is the core network responsible for executing motor commands and processing somatosensory information. Research indicates that the functional connectivity within the SMN may be weakened in children diagnosed with DCD, suggesting inefficient communication within the foundational network for sensorimotor integration (Rinat et al., 2020). Motor interventions, with a particular emphasis on sensorimotor integration, have been demonstrated to enhance functional connectivity between nodes within the SMN. This enhancement signifies the presence of a more seamless and coordinated information transmission within the brain’s sensorimotor system, thereby establishing the neural foundation for fluent and precise motor output.

In addition to the alterations in SMN-internal connectivity, the coupling relationship between the Default Mode Network (DMN) and task-positive networks (such as the SMN) is also subject to adjustment following intervention. The DMN demonstrates elevated levels of activity during periods of rest, with a suppression observed during cognitive activities related to external tasks. This negative coupling between the suppression of the default mode network (DMN) and task-related network activation (e.g., the sensorimotor network) is indicative of efficient brain task processing. It has been hypothesised that children diagnosed with Developmental Coordination Disorder (DCD) may demonstrate an inadequate capacity for default mode network (DMN) inhibition. Specifically, during motor tasks, the DMN maintains elevated activity levels, creating inappropriate competition with the SMN and disrupting task performance (McLeod et al., 2014). Following the implementation of motor intervention, this abnormal coupling may undergo enhancement, thereby facilitating more efficacious suppression of the DMN during tasks. This ensures that cognitive resources are more focused on the current motor task.

Furthermore, large-scale networks in healthy brains exhibit efficient “small-world” properties, maintaining high segregation between functionally specialised brain regions while achieving global integration (Brown-Lum and Zwicker, 2015). However, analyses reveal that the structural connectome of children with DCD shows weaker network segregation and integration, suggesting connective dysregulation (Caeyenberghs et al., 2016; Wilson et al., 2017a, 2017b). The present study hypothesises that motor intervention, defined as a complex sensorimotor experience, may drive optimization of brain network topology toward more mature and efficient patterns by promoting synchronised neuronal activity (Brown-Lum and Zwicker, 2017). This hypothesis is based on the principles of neuroplasticity.

### 4.2 Plasticity at the brain structural level

It is evident that, in addition to functional reorganization, the implementation of long-term, structured motor interventions has the capacity to induce substantial alterations in the anatomical structure of the brain. The utilisation of MRI-based structural imaging techniques, including voxel-based morphometry (VBM) and diffusion tensor imaging (DTI), facilitates the detection of this “hardware”-level plasticity.

#### 4.2.1 Grey matter changes: structural enhancement in key motor brain regions

VBM techniques have been demonstrated to facilitate quantitative analysis of differences in whole-brain grey matter volume, density, or thickness. Research conducted on children diagnosed with Developmental Coordination Disorder (DCD) has revealed grey matter structural abnormalities across multiple brain regions. These abnormalities have been shown to be directly correlated with motor and sensory deficits.

The sensorimotor cortex is a primary focus in this context. Children diagnosed with developmental coordination disorder (DCD) have been shown to exhibit reduced grey matter volume or density in the primary motor cortex and somatosensory cortex, which represent hand and body regions, in comparison to typically developing (TD) children (Reynolds et al., 2017; Malik et al., 2024a). These regions have been identified as the final output points for motor commands and the primary reception areas for sensory information. It has been demonstrated that structural abnormalities in these areas are directly linked to the motor clumsiness and perceptual deficits that are commonly observed in children diagnosed with Developmental Coordination Disorder (DCD).

Beyond the sensorimotor cortex, the cerebellum has also been the focus of significant research interest due to its abnormalities. Substantial evidence indicates the cerebellum plays a critical role in the pathophysiology of DCD (Subara-Zukic et al., 2022). Children diagnosed with developmental coordination disorder (DCD) frequently demonstrate a substantial decrease in cerebellar grey matter volume, particularly in the vermis and hemispheric regions implicated in motor learning, coordination, and timing functions. Furthermore, the volume of these regions has been shown to correlate positively with scores on the Movement Assessment Battery for Children, Second Edition (MABC-2), thereby directly confirming the association between grey matter reduction in cerebellar regions and impairments in motor learning and coordination (Zwicker et al., 2010; Gill et al., 2022).

Furthermore, the basal ganglia and thalamus, which are pivotal components within the motor circuitry, also play critical roles. As vital relay stations and regulators within the motor circuit, structural abnormalities in the grey matter of the basal ganglia (particularly the putamen) and thalamus are also associated with motor coordination difficulties in DCD.

Neuroimaging studies reveal that adolescents with DCD exhibit distinct activation patterns in the prefrontal cortex (e.g., dorsolateral prefrontal cortex, DLPFC) during motor tasks compared to typically developing peers. This finding is frequently interpreted as indicative of inadequate motor automation and excessive reliance on cognitive control (Al-Yahya et al., 2023). In line with this functional profile, structural MRI studies have also identified reduced grey matter volume in frontal regions (e.g., superior frontal gyrus, middle frontal gyrus) among children with DCD (Reynolds et al., 2017). This structural-functional covariation may result from neuroadaptive changes induced by prolonged reliance on cognitive effort to execute motor tasks, or may itself reflect inherent developmental vulnerability in the brains of individuals with DCD (Deng et al., 2014; Wilson et al., 2017a, 2017b).

Motor intervention has the capacity to address issues pertaining to grey matter structural abnormalities through the medium of environmental stimulation. As a potent environmental stimulus, it has the capacity to induce positive changes in grey matter structure by promoting synapse formation, dendritic branching, and neurogenesis (particularly in relevant brain regions). Although longitudinal VBM studies following DCD interventions remain nascent, research findings from athletes and children with other neurodevelopmental disorders provide reasonable grounds to infer the effects of targeted exercise training. It has been hypothesised that the intervention in question may have the capacity to halt or slow DCD-related delays or atrophy in grey matter development. Conversely, it has the capacity to stimulate increases in grey matter volume or density within critical motor brain regions, such as the cerebellum and sensorimotor cortex. This, in turn, establishes a robust structural foundation for functional enhancement. For instance, a complex task training study involving adolescents with DCD revealed significant post-training increases in grey matter density within the cerebellum and motor-related cortices. It is important to note that these structural changes were directly correlated with the degree of Behavioral improvement (Malik et al., 2024b). This provides preliminary direct evidence that lends support to the hypothesis that “movement can shape brain structure.”

#### 4.2.2 White matter changes: enhanced efficiency of neural conduction pathways

The brain’s white matter can be considered an “information superhighway,” composed of myelin-sheathed nerve fiber bundles that facilitate rapid signal transmission between distinct brain regions. Diffusion tensor imaging (DTI) technology indirectly assesses white matter microstructural integrity, including myelinisation levels and axonal integrity, by measuring the directional diffusion of water molecules within white matter fiber tracts, specifically the fractional anisotropy (FA) value. Higher FA values are generally indicative of superior structural integrity of white matter fibres and enhanced efficiency of neural signal transmission.

In children diagnosed with developmental coordination disorder (DCD), diffusion tensor imaging (DTI) studies have identified such abnormal white matter microstructural features (Brown-Lum et al., 2020). Specifically, reduced FA values were observed in multiple motor-related white matter pathways. Dysfunction in these pathways has been demonstrated to directly correlate with children’s motor coordination difficulties, primarily involving the following categories: The corticospinal tract, which functions as the primary descending pathway for transmitting motor commands from the brain to the spinal cord, demonstrates reduced fractional anisotropy (FA) values. This has been demonstrated to directly contribute to motor weakness and poor fine motor control in children diagnosed with Developmental Coordination Disorder (DCD). Reduced FA values in the corpus callosum (particularly in the anterior regions connecting bilateral motor cortices) may result in impaired bilateral limb coordination, thereby explaining why children with DCD often experience difficulties with tasks requiring bilateral hand coordination. The middle cerebellar peduncle (MCP) and inferior cerebellar peduncle (ICP) act as critical junctions for communication between the cerebellum and the cerebral cortex/spinal cord. It is hypothesised that microstructural abnormalities in these pathways may result in a weakening of the cerebellum’s role in coordinating, calibrating, and optimising motor commands. The superior longitudinal fasciculus (SLF), a vital pathway connecting the parietal, frontal, and temporal lobes, has been shown to participate in sensorimotor integration and spatial attention regulation (Jones et al., 2025). Its functional abnormalities may be closely related to the visuospatial processing deficits observed in children diagnosed with Developmental Coordination Disorder (DCD).

The efficacy of motor intervention in addressing the aforementioned white matter abnormalities is well-documented. The mechanism of action of this intervention is believed to be the promotion of myelin formation and the optimization of axonal integrity through targeted training. This, in turn, is thought to enhance the conduction efficiency of white matter pathways. Post-intervention DTI studies in children with DCD have provided preliminary evidence to support this hypothesis: following weeks to months of systematic training, significant increases in FA values were observed in key pathways such as the corticospinal tract and corpus callosum (Izadi-Najafabadi and Zwicker, 2021; Kangarani-Farahani and Zwicker, 2025). This finding suggests that motor intervention not only optimises functional activity across brain regions, but also structurally “reinforces” and “accelerates” communication pathways between brain areas. This synergistic interaction between structural and functional plasticity collectively establishes a robust neural foundation for improving motor behavior in children with DCD.

### 4.3 Integration of cognitive neurological mechanisms

The integration of evidence pertaining to brain function and structural plasticity facilitates the construction of a more comprehensive theoretical framework, thereby elucidating the mechanisms by which motor interventions engender therapeutic effects in cases of DCD. The present framework is predicated on two core concepts: internal model optimization and enhanced cognitive-motor coupling.

#### 4.3.1 Internal model optimization: from clumsy prediction to precise control

The “internal model” is a pivotal concept in contemporary motor control theory, denoting the neural representations formed within the brain concerning the dynamic interactions between the body and the external environment (Wolpert and Kawato, 1998). The model is comprised of two major components: the Forward Model (FM) and the Inverse Model (IM). The primary function of the FM is to predict the sensory consequences of issued movement commands, such as the final position of an arm. The IM is responsible for the calculation of the specific movement commands that are required to achieve the desired motion goals.

The core deficits of DCD are widely attributed to noise or insufficient precision within the internal model (Hyde and Wilson, 2011), directly leading to two critical issues. Firstly, impaired movement prediction results in an inability to accurately anticipate the outcomes of one’s actions, necessitating excessive reliance on slow online visual feedback to correct movements. This is indicative of a predominantly feedback-controlled approach rather than a smoother feedforward control. Secondly, difficulties in movement planning hinder the efficient translation of movement goals into the correct sequence of motor commands.

In order to address this fundamental issue, motor interventions—particularly those involving extensive repetitive practice with immediate feedback (e.g., task-oriented training, virtual reality games)—essentially constitute intensive sensorimotor integration training. It is posited that, through repeated cycles of “action-perception-comparison-correction,” the brain continuously calibrates its internal model. On the one hand, each successful or unsuccessful attempt provides data points that illustrate the relationship between “motor commands” and “actual sensory outcomes,” thereby accumulating substantial calibration material. Conversely, as practice advances, the brain employs this data to progressively reduce prediction errors in the internal model, thereby enhancing the accuracy of its anticipations of action outcomes.

From the perspective of neural mechanisms, this optimization process is achieved based on a series of functional and structural plasticity patterns, which mainly occur in the cerebellum and its associated networks. The cerebellum is widely regarded as the key neural basis for constructing internal models, and it continuously calibrates motor instructions by comparing the expected sensory outcomes with the actual feedback (i.e., error signals) (Ito, 2008).

From the perspective presented in this review, we can more systematically examine the neuroscientific evidence supporting the intervention-induced optimization of internal models. Firstly, at the functional level, post-intervention task-based functional magnetic resonance imaging studies show normalization of cerebellar activation and enhanced functional connectivity between the cerebellum and the sensorimotor cortex (Zwicker et al., 2011; Izadi-Najafabadi et al., 2022). This indicates that the “comparator” of the cerebellum operates more efficiently and communicates more effectively with the motor execution regions. Secondly, this functional improvement is based on structural enhancement. The increase in grey matter volume within the cerebellum after intervention (Malik et al., 2024b) may provide a more powerful neural infrastructure for internal model calculations. Thirdly, the communication lines themselves have also been upgraded. The improvement in the microstructure integrity of white matter pathways (such as the midbrain and inferior cerebellar peduncles and corticospinal tracts) that are crucial for cerebellar communication (Izadi-Najafabadi and Zwicker, 2021). This enhanced structural connectivity ensures that the precise predictions generated by the cerebellum can be rapidly and accurately transmitted to the motor cortex and the spinal cord.

Therefore, the assumption that “intervention optimises cerebellar function to refine internal models” is not supported solely by individual research results, which indicates the coordinated functional recovery, structural growth, and connection enhancement patterns within the cerebellum-thalamus-cortex network. This multi-level plasticity enables the brain to adopt more precise feedforward control, reduces the need for slow feedback-based corrections, and ultimately manifests as smoother, more accurate, and more automatic motor behaviors in children with developmental coordination disorders.

#### 4.3.2 Enhancing cognitive-motor coupling: from effortful control to strategic application

The excessive prefrontal activation observed in children diagnosed with Developmental Coordination Disorder (DCD) during motor tasks reveals a tense rather than synergistic relationship between their cognitive and motor systems. Before intervention, these coupling exhibits inefficiency, with substantially higher-order cognitive resources being consumed by basic motor control, resulting in “cognitive overload.”

Cognitive strategy interventions such as CO-OP have been demonstrated to possess a unique value by means of consciously and systematically reshaping this cognitive-motor coupling. In contrast to the mere reduction of cognitive engagement, the pedagogical approach under scrutiny involves the instruction of children in the application of cognitive resources more intelligently and strategically. This involves a transition from unconscious compensation to conscious strategy. Before intervention, DCD children’s reliance on the prefrontal cortex may signify an unconscious, passive neural compensation. CO-OP has been developed to facilitate the active and controlled application of cognitive strategies. It has been demonstrated that children learn to utilise a metacognitive framework involving the processes of goal planning, execution, and checking. This framework encompasses the planning of actions before movement, the monitoring of actions during execution, and the reflection upon actions following the completion of the action.

This active cognitive engagement has been demonstrated to optimise resource allocation, with research indicating a shift in prefrontal activity from “micromanaging basic movement details” to “focusing on higher-level goal setting, problem solving, and strategy adjustment.” In other words, cognitive resources transition from a state of passive ‘compensation’ to active ‘management’. This phenomenon can be explained by the hypothesis that, as skills become automated, there is a possibility of a decrease in overall prefrontal activation. However, this is counterbalanced by a greater degree of precision and effectiveness in activation at critical decision points.

From a neurological perspective, this is characterised by reconfigured functional connectivity between the prefrontal cortex and other brain regions. Following the intervention, the connections between the prefrontal cortex and motor subsystems, such as the basal ganglia and cerebellum, may become more efficient, resulting in the formation of a synergistic “cognitive-motor joint network.” Concurrently, as previously mentioned, the prefrontal cortex’s regulatory influence over the Default Mode Network (DMN) may also be amplified, thereby ensuring task-specific focus. This enhanced, more efficient cognitive-motor coupling is not only crucial for motor skill improvement but may also serve as the neural basis for transferable enhancements in executive functions (e.g., working memory, cognitive flexibility).

In summary, the present study demonstrates that motor intervention can induce multi-level evidence of brain plasticity in children diagnosed with Developmental Coordination Disorder (DCD). From a functional perspective, the intervention prompts a transition from inefficient, consciously controlled patterns to automated, efficient modes that are characterised by the dominance of core motor circuits. Concurrently, it enhances overall connectivity and efficiency across brain networks. Structurally, it has been hypothesised that this may promote the development of grey matter regions associated with motor learning and execution, and strengthen the integrity of white matter tracts connecting these areas. Collectively, these changes support two core cognitive-neural mechanisms: The terms ‘internal model optimization’ and ‘enhanced cognitive-motor coupling’ are employed.

These findings carry profound clinical implications. It has been demonstrated that motor interventions for DCD extend beyond the mere instruction of movements; rather, they specifically “remodel the brain,” thereby promoting its neurodevelopment toward a more optimal trajectory. This provides the most fundamental biological basis for the legitimacy of motor intervention and underscores the importance of early, active intervention during childhood, when brain plasticity is highest. Future research should involve meticulously designed longitudinal neuroimaging studies to directly track brain change trajectories during intervention and distinguish the specific neuroplasticity patterns potentially induced by different intervention methods. This will establish the foundation for the development of more precise and efficient personalized neuro-rehabilitation protocols.

## 5 Constructing an integrated model of “behavioral intervention-neural remodeling-functional improvement”

The preceding discussion has clearly delineated a causal chain, commencing from external Behavioral and motor interventions, progressing to internal neural remodeling processes, and culminating in functional improvement in individuals. The core objective of this paper is to synthesise these fragmented research evidences into a comprehensive theoretical model, further analyzing its scientific value and practical implications. The central argument of this model is that neuroplasticity serves as the intrinsic biological foundation for how movement interventions drive Behavioral improvements in children with DCD. The potential for diverse intervention methods to contribute to this transformative process is predicated on their capacity to act on either shared or specific neural pathways.

### 5.1 Development of the “motor intervention-neural remodeling-functional improvement” integration model

**Figure 1:**
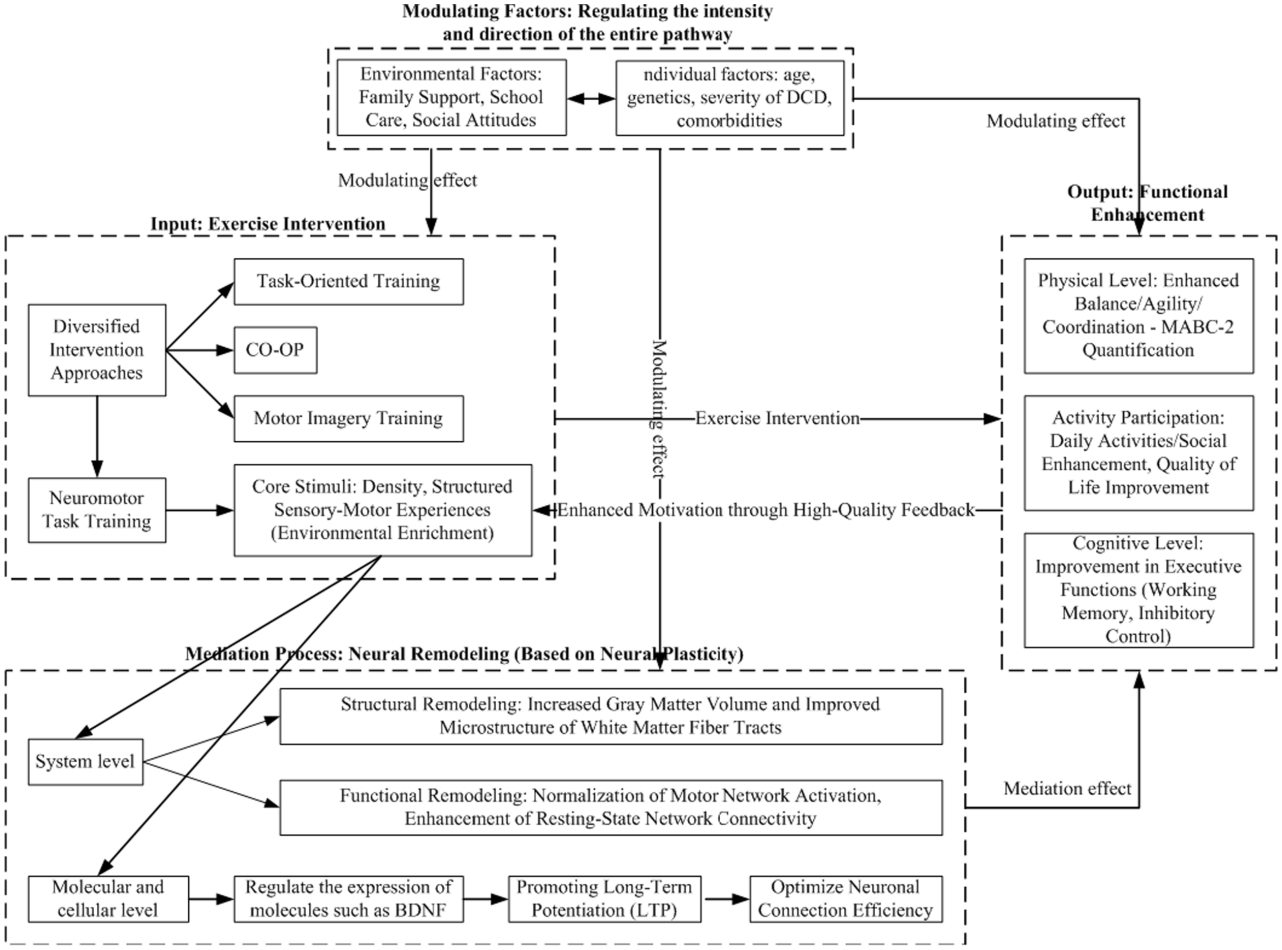
Integrated model of exercise intervention-neural remodeling-functional improvement.

Drawing upon extant literature, a multi-level, dynamic integration model (see Figure 1) has been formulated to illustrate the specific pathways through which motor interventions function in the rehabilitation process of children diagnosed with DCD.

The integrated model of motor intervention, neuroplasticity, and functional improvement is comprised of three primary components. The first is motor intervention (input), which serves as the logical starting point of the model. Intervention methods demonstrate a high degree of diversity, as evidenced by the utilisation of diverse approaches such as task-oriented training, CO-OP (Cognitive Oriented Movement Program), motor imagery training, and neuromotor task training. A salient commonality among these methods is the provision of intensive and structured sensorimotor experiences and learning opportunities to children. These experiences constitute the “environmentally enriched” stimuli that drive adaptive changes in the brain. The second component is neuroplasticity (mediating process), which is the core mechanism of the model. As an external trigger, motor intervention leverages neuroplasticity to induce multi-level adaptive changes in the brain, spanning from microscopic molecular to macroscopic systemic levels. At the molecular and cellular levels, interventions regulate the expression of key molecules such as brain-derived neurotrophic factor (BDNF), enhancing synaptic plasticity (e.g., long-term potentiation, LTP) to optimise neural signalling and connectivity efficiency (Kleim and Jones, 2008). These microscopic alterations establish the biological foundation for more extensive brain remodeling.

At the systems level, such changes manifest primarily in two aspects: Firstly, there is functional remodeling, which specifically involves the normalization of motor network activation patterns during task states. This includes a reduced reliance on the prefrontal cortex and enhanced cerebellum-basal ganglia function, alongside improved connectivity efficiency within resting-state brain networks (e.g., the sensorimotor network and the default mode network). Secondly, there is structural reorganization, manifested as a potential increase in grey matter volume or density in motor learning-related regions (e.g., the sensorimotor cortex and the cerebellum), accompanied by improved microstructural integrity of white matter tracts connecting these areas (e.g., the corticospinal tracts and the corpus callosum). Thirdly, functional improvement (output) is the external manifestation of neural remodeling. The immediate effect of neural remodeling is a substantial enhancement of the brain’s information processing capacity. This optimization is further externalized into functional improvements across three levels: At the motor level, the most direct improvements focus on motor skills themselves, such as enhanced balance, fine manual dexterity, and overall motor coordination. The quantitative assessment of these improvements can be facilitated by the utilisation of standardised tools such as the MABC-2 (Movement Assessment Battery for Children, Second Edition). At the activity and participation level, enhanced motor abilities enable children to engage more smoothly in daily activities, such as writing, dressing independently, and participating in school physical education classes. These abilities also boost their confidence and opportunities for social interaction and play.

This, in turn, results in an enhancement in the overall quality of life. At the cognitive level, given the close interaction between motor and cognitive systems, the neuroplasticity triggered by motor interventions—particularly enhanced prefrontal cortex function and improved cognitive-motor coupling efficiency—may also produce transfer effects. These can enhance children’s executive functions, including working memory capacity and inhibitory control abilities.

It is imperative to emphasise that this model does not represent a unidirectional linear process. Functional enhancements exert a reciprocal influence on the experiential facets of the intervention. For instance, the success of the intervention is known to enhance children’s motivation to participate, encouraging more active engagement. Concurrently, these enhancements furnish superior sensorimotor feedback, thereby facilitating subsequent neural remodeling in the brain. This process ultimately establishes a favourable self-reinforcing cycle. Furthermore, individual factors (e.g., the child’s age, genetic background, severity of DCD symptoms, and comorbid conditions) and environmental factors (e.g., the level of family support and societal awareness and acceptance of DCD) jointly modulate the intensity and developmental trajectory of the entire intervention-remodeling-improvement process.

### 5.2 Neuroplasticity: a unified biological framework for behavioral improvement

This comprehensive model posits that neuroplasticity is not merely a related outcome but is a crucial biological foundation upon which the therapeutic effects of behavioral interventions rely. This section does not repeat the specific imaging findings detailed in Chapter 4, but aims to elucidate the theoretical role of neuroplasticity within this model.

Firstly, neuroplasticity provides a unified explanation for the effectiveness of various intervention methods. Whether it is a task-oriented intervention approach (such as NTT), a cognitive strategy-based intervention approach (such as CO-OP), or an intervention approach leveraging technology (such as VR), their common core lies in providing structured, high-intensity, and meaningful perceptual-motor experiences (Brown-Lum et al., 2020). These experiences serve as key environmental stimuli, activating the brain’s inherent plasticity mechanisms—strengthening synaptic connections, promoting myelination, and optimising functional networks—to “shape” the developing brain (Zwicker et al., 2011; Patel et al., 2013). For instance, Biotteau et al. (2016) demonstrated that children exhibiting more pronounced enhancement in cerebellar-cortical connectivity showed more significant increases in the automation level of their motor skills. Therefore, although the behavioral techniques vary, they all point to the same fundamental biological process: guiding the brain toward a more efficient and adaptive organisational pattern. This perspective shifts the focus from debating “the best” technology to how to best stimulate neuroplasticity to adapt to each child’s specific circumstances.

Secondly, the neuroplasticity framework emphasizes the crucial importance of early intervention. Childhood is a stage of enhanced neuroplasticity, often described as the “sensitive period” for motor and cognitive development. During this period, the brain has a unique receptivity to changes in dependence on experiences (Kolb and Gibb, 2011). Early targeted intervention in DCD can take advantage of this enhanced plasticity, guiding neural development onto a more normal trajectory, thereby potentially avoiding the solidification of adverse neural patterns and a series of subsequent psychological and social consequences. In contrast, later intervention is still valuable, but it may require more intensive or longer input to achieve similar results, as the plasticity potential of the brain decreases. As posited by Al-Yahya et al. (2023), the developing brain has been shown to exhibit greater responsiveness and adaptability to experiential stimuli in comparison to the adult brain. Therefore, this model emphasizes that “the timing of intervention” is just as biologically significant as “the content and method of intervention” and “the way of intervention.”

### 5.3 Neural pathways of different intervention methods: shared and specific mechanisms

Based on the unified role of neural plasticity, this section will explain how the diversity of intervention methods (the “input” layer of our model) can be understood through its utilisation of common and specific neural pathways (part of the “mediating process” layer). This section is not intended to repeat all the neuroimaging evidence, but rather to illustrate how different inputs can lead to the same functional output through different pathways within the brain.

#### 5.3.1 Shared neural pathways: the common Foundation for Intervention Efficacy

All effective exercise interventions essentially strengthen a set of core neural circuits that form the foundation of motor control. These include: (1) the primary sensory-motor integration circuit (somatosensory-motor cortex), which is the basis for linking perception with action; (2) the cerebellum-thalamus-cortex circuit, which is crucial for precisely adjusting the rhythm, strength, and coordination of movements; (3) for bilateral tasks, the corpus callosum, which helps facilitate information exchange between the two hemispheres of the brain. Strengthening these common pathways is an important foundation for any motor benefit.

#### 5.3.2 Specific neural pathways: unique targets of intervention methods

Specific paths: “imprints” of different methods. Each intervention method’s unique “characteristics” stem from its different emphasis on specific brain networks.

*Task-oriented and neuro-motor training (NTT)*: Through repetitive and functional task practice, these methods focus on the sensory-motor cortex, cerebellum, and basal ganglia. Their main effect is to optimise the “internal model” of the brain for specific actions, shifting the control from conscious, effortful processing to more automatic execution (Ferguson et al., 2013).

*Cognitive-oriented daily performance (CO-OP)*: This method emphasizes the application of metacognitive strategies (i.e., “goal–plan–execute–check”), significantly activating the prefrontal cortex (PFC), especially its dorsolateral part. Its neural feature is the enhancement of “cognitive-motor coupling,” training the brain not only to know what to do but also to learn how to handle and solve motor problems in a strategic manner. This makes the activation of the prefrontal cortex more efficient and improves the top-down regulation of the motor network (Schwartz et al., 2020; Izadi-Najafabadi et al., 2022).

*Motor imagery (MI) and action observation (AO)*: These two methods utilise the mirror neuron system (MNS), including the inferior parietal lobe and premotor cortex. They strengthen the neural representation of actions without performing actual movements, thereby enhancing motor planning, imitation ability, and the vividness of motor imagery (Mulder, 2007; Ertelt et al., 2007; Wilson et al., 2016; Reynolds et al., 2015).

*Aerobic exercise*: This type of exercise provides more extensive “nutrients” for brain health, promotes the release of neurotrophic factors, and improves cerebral vascular function. It creates a more relaxed and favourable physiological environment, enhancing the brain’s plasticity ability, thereby improving the effectiveness of intervention (Thomas et al., 2016).

In summary, this comprehensive model takes into account both integration phenomena and differentiation phenomena. Different intervention methods, through their unique mechanisms of action, combine the common basic network with specific neural targets.

### 5.4 The regulatory role of comorbidities on neural plasticity pathways

The comprehensive model shown in Figure 1 must also take into account the influence of the comorbidity phenomenon described in Section 2.2. When there is a comorbidity situation, the neural pathways affected by the intervention measures may undergo fundamental changes. For example, in children with Developmental Coordination Disorder (DCD) and Attention Deficit Hyperactivity Disorder (ADHD), the low activity of the prefrontal cortex related to ADHD may interact with the low efficiency of the prefrontal cortex in motor tasks observed in DCD. Interventions aimed at promoting the coupling of prefrontal cognitive-motor functions, such as CO-OP, may produce different plasticity patterns from those of children with DCD. During the intervention process, attention regulation issues are addressed first, followed by the learning of effective strategies. Similarly, in children with DCD and Autism Spectrum Disorder (ASD), the abnormalities of the mirror neuron system (MNS) and social brain networks. This makes them more likely to activate different circuits in intervention measures related to action observation or imitation. Therefore, the general and specific neural pathways discussed earlier are not fixed; they are dynamically regulated by the dynamic characteristics of the complete neurobiology of children. This indicates that future research must abandon the single diagnostic model and systematically explore the impact of comorbidity on the intervention process.

### 5.5 From evidence to practice: the framework for clinical decision-making

By integrating the evidence on intervention effects (Chapter 3) and the neural pathways involved in these interventions (Section 5.3), we can propose a preliminary framework to guide clinical decisions. This framework aims to abandon the “one-size-fits-all” approach and instead match intervention strategies based on the specific clinical conditions and main functional impairments of children (see Table 1).

**Table 1:** A guide to matching intervention strategies to clinical profiles in DCD.

Primary functional impairment/clinical profile	Recommended intervention approach	Targeted neural pathway/mechanism	Key considerations for implementation
Deficits in fine motor skills and executive function (e.g., poor handwriting, difficulty with multi-step tasks)	CO-OP (Cognitive Orientation to daily Occupational Performance)	Prefrontal Cortex (cognitive-motor coupling), metacognitive strategy use	Requires good verbal comprehension and motivation. May need adaptation for children with significant attention deficits.
Poor gross motor coordination and balance (e.g., awkward gait, difficulty riding a bike)	NTT (Neuromotor Task Training) or Task-oriented training	Sensorimotor cortex, cerebellum, basal ganglia (internal model optimization)	Emphasizes intensive, repetitive practice of functional tasks with graded difficulty.
Impaired motor planning and imagery (e.g., difficulty imitating actions, poor performance on mental rotation tasks)	Motor Imagery (MI) and Action Observation (AO) training	Mirror Neuron System (MNS), premotor cortex, inferior parietal lobule	Can be a useful adjunct or starting point for children with severe motor execution difficulties.
Low motivation, poor engagement, and need for safe practice environment	Virtual Reality (VR)/Active Video Games (AVG)	Sensorimotor networks (via engaging, repetitive practice)	Should be used as a supplement to, not a replacement for, real-world functional task practice. Monitor movement quality.
Significant physical deconditioning and low activity levels	Aerobic and Fitness training combined with skill-based intervention	Global brain health (neurotrophins, vascular function)	Provides a supportive physiological foundation for more targeted skill learning.
DCD with comorbid ADHD	CO-OP + Behavioral and attention regulation strategies	PFC (cognitive control) + DMN/SMN coupling	Simplify instructions, use shorter sessions, provide external rewards, and integrate movement breaks.
DCD with comorbid ASD	Task-oriented training with structured, predictable environment + explicit social stories if in groups. MI/AO may be less effective if MNS dysfunction is suspected.	Sensorimotor networks (core) with adaptations for social-communication challenges.	

This Strategies is merely an illustrative tool for clinicians. The selection of intervention measures should always be a collaborative process involving children, families, and therapists, taking into account the goals, preferences of the children, and the background of the families. Moreover, as our understanding of the neural mechanisms underlying different subtypes of developmental coordination disorders and their comorbidities continues to deepen, this Strategies can be improved and verified through future research, thus moving closer to achieving the goal of truly personalized and precise rehabilitation.

## 6 Research limitations and future prospects

Despite the existence of research that offers encouraging evidence for comprehending the impacts of motor interventions on the behavior and neuroplasticity of children diagnosed with Developmental Coordination Disorder (DCD), it is important to acknowledge that this domain is still in its nascent stages of development and is confronted with a multitude of methodological and theoretical limitations. It is imperative to acknowledge these challenges and formulate future research directions accordingly to facilitate progress in scientific research and clinical translation in this domain.

### 6.1 Limitations of current research

#### 6.1.1 Methodological limitations: sample size, design, and causal inference

Most current neuroimaging studies suffer from small sample sizes (Adams et al., 2014; Biotteau et al., 2016). Small-sample research not only lacks statistical power to detect moderate or small effects but also faces challenges in replicability and generalizability of findings. More critically, many studies lack an active control group (Goulardins et al., 2017). An ideal control group should receive an intervention matching the experimental group in intensity, attention, and expected effects but differing in content (e.g., general art activities or routine instruction). This design is essential to exclude non-specific factors (such as additional attention or the Hawthorne effect) from influencing observed behavioral improvements and neural changes. The absence of such control groups makes it difficult to unequivocally attribute observed effects to specific components of the intervention itself, weakening the strength of causal inferences.

#### 6.1.2 Insufficient depth in behavior-neural correlation analysis

While some studies have begun reporting correlations between behavioral improvements and neural changes post-intervention, in-depth mediation analyses remain scarce (Zwicker et al., 2011). Mediation analysis is a critical statistical method for testing the core hypothesis that “neural plasticity is the intrinsic mechanism underlying behavioral improvement.” It requires demonstrating: (a) the intervention significantly impacts behavior (path c); (b) the intervention significantly affects neural measures (path a); (c) after controlling for neural measures, the intervention’s effect on behavior weakens or disappears (path c’), while neural measures themselves significantly predict behavior (path b). Current research mostly stops at the first two steps, lacking testing of the complete mediation model. Therefore, it remains inconclusive whether neural remodeling is a “mediating variable” for behavioral improvement or merely a “correlated byproduct.”

#### 6.1.3 Evidence gap regarding long-term efficacy and persistence of neuroplasticity

The vast majority of intervention studies are short-term, typically lasting 8–12 weeks, with assessments conducted only immediately after intervention completion (Ferguson et al., 2013). Long-term follow-up studies on intervention effects are extremely scarce. This leaves several critical questions unresolved: Can improvements in motor skills and daily participation be sustained long-term? Are the neuroplastic changes induced by intervention temporary or enduring? Does the brain “revert” to its original activity patterns when the intervention ceases? If changes are enduring, do they reflect the consolidation of neural connections? If changes are transient, what “maintenance dose” or “reinforcement intervention” is needed to consolidate gains? Answers to these questions are crucial for developing clinical interventions with long-term benefits.

#### 6.1.4 Neglect of individual differences and heterogeneity

Significant heterogeneity exists within the DCD population, manifesting in motor impairment severity, co-occurring cognitive deficits (e.g., executive function difficulties), comorbid conditions (e.g., ADHD, ASD), and etiology (Blank et al., 2019). However, existing research predominantly treats DCD as a homogeneous group for analysis, rarely exploring how these individual differences influence responses to specific interventions (i.e., moderation effects). This approach may lead to biased estimates of overall intervention effectiveness and fails to address the core clinical question: “Which intervention is most effective for DCD children with specific characteristics?”

### 6.2 Future research directions for DCD

To overcome the aforementioned limitations and advance the field, future research should focus on the following cutting-edge directions.

#### 6.2.1 Toward precision rehabilitation: integrating multimodal biomarkers and addressing comorbidity

Future research should adopt the concept of precision medicine to achieve “precision rehabilitation” in DCD disorders. This means abandoning the “one-size-fits-all” approach and instead implementing personalized treatment by identifying the best intervention measures and tailoring the most effective intervention plans based on individual characteristics. To achieve this goal, it is necessary to integrate multiple levels of biomarkers and systematically consider the complexity of comorbidities.

Integrate multimodal biomarkers. For instance, genetic biomarkers can explore candidate genes related to neuroplasticity and motor learning (such as brain-derived neurotrophic factor, catecholamine transporter polymorphisms) to determine whether they can predict differences in individual responses to intervention measures. Neuroimaging biomarkers can use baseline (before intervention) brain structure and functional features as predictive indicators. For example, do children with excessive activation in the prefrontal cortex at baseline respond better to intervention measures emphasizing cognitive strategies (such as CO-OP)? Children with weak brain-basal ganglia circuits may require more basic motor training tasks? Behavioral and cognitive biomarkers can incorporate detailed cognitive neuropsychological assessments (such as executive function, working memory, sensory-motor integration) into the predictive model. Through large-scale, multi-center collaborations, establish a comprehensive database integrating genetic, neuroimaging, behavioral and clinical data, and apply advanced statistical methods such as machine learning to build a model that can effectively predict individualized intervention responses.

View comorbidity issues as key regulatory factors. Developmental coordination disorder (DCD) often co-occurs with attention deficit hyperactivity disorder (ADHD), autism spectrum disorder (ASD), and other neurodevelopmental disorders. However, most intervention studies treat DCD as a unified category and often exclude children with comorbid conditions. This creates a critical evidence gap: it is currently unclear whether there are differences in intervention effects and the underlying neuroplasticity mechanisms between pure DCD patients and those with comorbidity characteristics. Future research must prioritize systematic studies on comorbidity issues and consider them as key regulatory factors in intervention outcomes. Key questions include: Does comorbidity status affect the neuroplasticity caused by intervention? Should intervention strategies be adjusted for specific comorbidity characteristics (such as using attention regulation strategies for DCD + ADHD cases, or using structured visual support for DCD + ASD cases)? How should assessment and intervention plans be adjusted according to the actual clinical complexity? To address these issues, we recommend using stratified sampling for prospective recruitment, conducting *post hoc* analyses of subgroups in existing trials, and developing customized intervention plans for specific comorbidity situations. It is crucial that comorbidity status be included in the predictive model along with genetics, neuroimaging and cognitive biomarkers, while recognizing that the complete neurobiological characteristics of children (including concurrent conditions) are essential for accurate intervention matching.

By combining multimodal biomarkers with systematic attention to comorbidity, future research can go beyond the traditional “pure developmental coordination disorder” model and move toward truly personalized precision rehabilitation: providing appropriate interventions to the right children at the right time.

#### 6.2.2 Exploring mechanistic interventions: combining non-invasive brain stimulation with exercise

To more actively guide neuroplasticity, future research may explore integrating non-invasive brain stimulation (NIBS)—such as transcranial direct current stimulation (tDCS) or transcranial magnetic stimulation (TMS)—with behavioral interventions. The underlying principle is that NIBS can temporarily modulate excitability in specific brain regions (e.g., enhancing motor cortex excitability with anodal tDCS), thereby creating a “primed” brain state. During this state, motor training may yield synergistic effects, significantly enhancing neuroplasticity and behavioral improvement (Lefebvre and Liew, 2017). For instance, applying tDCS to the primary motor cortex or prefrontal cortex while children perform motor imagery or task-oriented training could test whether this combined intervention yields more pronounced and rapid effects than behavioral intervention alone. This not only explores a potential therapeutic enhancement but also provides direct experimental validation of the causal relationship between “behavior and neuroplasticity.”

#### 6.2.3 Driving technological and methodological innovation: ecological validity and objective assessment

Future research should actively adopt new technologies to enhance ecological validity and assessment objectivity. High Ecological Validity Neurotechnologies: Functional near-infrared spectroscopy (fNIRS) and portable electroencephalography (EEG) devices enable real-time monitoring of children’s brain activity during authentic tasks in natural settings (e.g., classrooms, therapy rooms). This overcomes the limitations of traditional fMRI environments, offering a novel window into understanding “online” neural processing. Digital Phenotyping: Wearable sensors (e.g., accelerometers, gyroscopes) and video-based motion capture technologies enable long-term, objective quantification of children’s physical activity levels, movement patterns, and quality within natural living environments (Smits-Engelsman et al., 2015). This vast objective data serves as a “digital biomarker” for intervention efficacy, reflecting real-world changes in children’s daily lives more accurately than traditional questionnaires or laboratory tests.

#### 6.2.4 Expanding the lifespan perspective: focusing on adolescent and adult DCD

The vast majority of research centers on school-aged children, with severe gaps in intervention studies and neurobiological mechanism exploration for adolescents and adults with DCD. However, DCD is a lifelong condition, and the challenges it presents during adolescence and adulthood—such as driving, career choices, and mental health issues—may be more complex and profound (Cairney, 2020). Future research urgently requires: developing age-appropriate interventions for adolescents that address career readiness, social interaction, and independent living skills; investigating the efficacy and neural mechanisms of motor interventions in alleviating comorbid mental health issues like anxiety and depression among adolescents and adults with DCD; and exploring whether adult brain plasticity can still provide a biological basis for effective interventions, thereby offering scientific support for the “never give up” rehabilitation philosophy. Current research on the neural mechanisms of motor interventions for DCD has outlined a promising blueprint, yet it faces significant challenges in sample representativeness, strength of causal inference, depth of mechanism validation, and long-term perspective. Future research pathways should be more ambitious. Through large-scale collaborative studies, integration of multimodal biomarkers, application of advanced technologies, and a lifespan perspective, this blueprint can be progressively transformed into a precise roadmap. This will ultimately achieve the grand goal of tailoring highly effective rehabilitation strategies for each DCD individual, thereby genuinely improving their quality of life.

## 7 Conclusion

This paper systematically reviews existing literature to construct an integrated framework demonstrating how exercise interventions improve functional outcomes in children with DCD through neuroplasticity mechanisms. In consideration of the extant evidence, the following core conclusions are posited: Structured motor interventions have been demonstrated to be efficacious in enhancing external Behavioral outcomes, including motor skills, activity participation, and quality of life, in children diagnosed with DCD. Furthermore, these interventions serve as potent environmental stimuli, inducing positive neuroplastic changes in both brain function and structure. These changes are closely linked to Behavioral improvement. This insight denotes a shift in the prevailing paradigm of DCD rehabilitation, transitioning from the erstwhile “black box” model that placed chief emphasis on Behavioral remediation to a “white box” model that explores the underlying neurobiological mechanisms. This development signifies a pivotal progression in the realm of DCD rehabilitation practice, transitioning from an “experience-based” approach to a more robust and “science-based” methodology. The hypothesis that viewing motor intervention as a potent tool capable of effectively guiding beneficial brain plasticity in children with DCD opens promising new avenues for understanding and improving this neurodevelopmental disorder is one that merits further investigation. The systematic and in-depth revelation of the intrinsic patterns of interaction between interventions and the brain is of significant theoretical value and provides an indispensable theoretical foundation for the development of more targeted, efficient, and personalized DCD rehabilitation programmes in the future. The overarching objective is to measurably enhance the life trajectories of children with DCD through neuroscience-guided precision interventions, thereby empowering them to actualise their full potential.
